# *Mycobacterium abscessus* Morphotype Comparison in a Murine Model

**DOI:** 10.1371/journal.pone.0117657

**Published:** 2015-02-12

**Authors:** Lindsay J. Caverly, Silvia M. Caceres, Cori Fratelli, Carrie Happoldt, Kelley M. Kidwell, Kenneth C. Malcolm, Jerry A. Nick, David P. Nichols

**Affiliations:** 1 Department of Pediatrics, University of Michigan, Ann Arbor, Michigan, United States of America; 2 Department of Medicine, National Jewish Health, Denver, Colorado, United States of America; 3 Department of Pediatrics, National Jewish Health, Denver, Colorado, United States of America; 4 Department of Biostatistics, University of Michigan, Ann Arbor, Michigan, United States of America; 5 Department of Medicine, University of Colorado Denver, Aurora, Colorado, United States of America; The Hospital for Sick Children and The University of Toronto, CANADA

## Abstract

Pulmonary infections with *Mycobacterium abscessus* (*M. abscessus*) are increasingly prevalent in patients with lung diseases such as cystic fibrosis. *M. abscessus* exists in two morphotypes, smooth and rough, but the impact of morphotype on virulence is unclear. We developed an immune competent mouse model of pulmonary *M. abscessus* infection and tested the differences in host inflammatory response between the morphotypes of *M. abscessus*. Smooth and rough morphotypes of *M. abscessus* were isolated from the same American Type Culture Collection strain. Wild type and cystic fibrosis mice were intratracheally inoculated with known quantities of *M. abscessus* suspended in fibrin plugs. At the time of sacrifice lung and splenic tissues and bronchoalveolar lavage fluid were collected and cultured. Bronchoalveolar lavage fluid was analyzed for leukocyte count, differential and cytokine expression. Pulmonary infection with *M. abscessus* was present at both 3 days and 14 days post-inoculation in all groups at greater levels than systemic infection. Inoculation with *M. abscessus* rough morphotype resulted in more bronchoalveolar lavage fluid neutrophils compared to smooth morphotype at 14 days post-inoculation in both wild type (p = 0.01) and cystic fibrosis (p<0.01) mice. Spontaneous *in vivo* conversion from smooth to rough morphotype occurred in 12/57 (21%) of mice. These mice trended towards greater weight loss than mice in which morphotype conversion did not occur. In the described fibrin plug model of *M. abscessus* infection, pulmonary infection with minimal systemic dissemination is achieved with both smooth and rough morphotypes. In this model *M. abscessus* rough morphotype causes a greater host inflammatory response than the smooth based on bronchoalveolar lavage fluid neutrophil levels.

## Introduction

Nontuberculous mycobacteria (NTM) are environmental organisms ubiquitous in soil and water. Pulmonary NTM infections primarily affect individuals with underlying lung diseases such as chronic obstructive pulmonary disease, bronchiectasis and, especially, cystic fibrosis (CF)[1]. Prevalence of pulmonary NTM infections has increased over recent decades[2,3], with prevalence of NTM infection in CF patients in the United States currently reported at 11–13%[4,5]. Of the NTM, *Mycobacterium avium* complex and *Mycobacterium abscessus* complex (*M*. *abscessus*) are the species most commonly recovered from CF airways[4,5]. *M*. *abscessus* is a rapidly-growing NTM and is widely considered to be the most pathogenic of the NTM infecting CF patients due to its multidrug resistance[6], poor response to treatment[7] and association with decline in lung function[5]. But interestingly, *M*. *abscessus* pulmonary infections are associated with a wide clinical spectrum of disease in CF patients, ranging from asymptomatic, transient colonization to significant lung function decline[5,8,9], thus making it often difficult to decide when to initiate *M*. *abscessus* treatment. It is unclear which *M*. *abscessus* virulence factors may contribute to more severe disease.

Colony morphotype is a potential *M*. *abscessus* virulence factor that may contribute to disease severity. *M*. *abscessus* exists in two distinct morphotypes, smooth and rough, that differ in their gross colony appearances when grown on solid media due to their differing amounts of cell wall glycopeptidolipids[10,11]. Spontaneous conversion between the morphotypes occurs at a rate of ~ 1 in 10^6^ *in vitro*[11], and both morphotypes have been recovered from the human airways[12–15]. It has been proposed that the smooth morphotype of *M*. *abscessus* initially colonizes the airways and forms biofilms, with subsequent *in vivo* conversion to the rough morphotype leading to more invasive disease[11]. Limited human data supports the hypothesis of increased pathogenicity from the rough morphotype. The rough morphotype is associated with chronic colonization in the CF airways[12], and case reports describe dramatic clinical declines and/or death in CF patients with *M*. *abscessus* rough morphotype in their airways[13,16]. However, differentiating between the morphotypes of *M*. *abscessus* is not currently performed in most clinical laboratories, as the clinical utility of doing so is not clear.

Prior mouse models of *M abscessus* pulmonary infection have demonstrated increased pathogenicity *in vivo* from the rough compared to the smooth morphotype[10,11]. These prior models, however, have been limited by the difficulty in establishing a persistent pulmonary infection with minimal systemic spread in immune competent animals. In the earliest *in vivo* study of *M*. *abscessus* morphotype differences, intratracheal inoculation with the smooth morphotype of *M*. *abscessus* was quickly cleared from the lungs of immune competent mice[10]. Persistent *M*. *abscessus* infection was achieved with both rough and smooth morphotypes through use of immune deficient (SCID) mice. These mice had greater persistence of both pulmonary and systemic infection with the rough compared to the smooth morphotype[10], a finding which was confirmed by a more recent model that used an intranasal inoculation of *M*. *abscessus*[11]. Aerosol delivery of *M*. *abscessus* created persistent pulmonary infection in immune competent mice, but in this model systemic infection based on splenic cultures was similar to that in the found in the lung[17]. More recently, an aerosol delivery of *M*. *abscessus* was used to create a pulmonary infection in immune competent mice, but the morphotype of the *M*. *abscessus* was not described [18].

Similarly, intravenous (IV) *M*. *abscessus* infection models have found greater mortality and higher levels of TNF-α with the rough compared to the smooth morphotype in immune competent (C57BL/6) mice[19]. However, in addition to the differences in immune response to inhaled versus IV delivery of mycobacterial infection[18,20] 20, IV infection models have also been limited by their creation of predominately systemic, rather than pulmonary, infection[18,21]. These prior models have had significant limitations in their applicability to human NTM pulmonary infections in diseases such as CF, largely due to their use of immune deficient animals and/or predominance of systemic infection over pulmonary. An immune competent animal model of pulmonary *M*. *abscessus* infection would be useful for *in vivo* comparisons of the smooth and rough morphotypes to help better define the role of morphotype in *M*. *abscessus* pathogenicity.

We describe a novel mouse model of *M*. *abscessus* infection in which intratracheal inoculation of *M*. *abscessus* suspended in thrombin and fibrinogen solutions traps the bacteria in the distal airways in viscous fibrin plugs. With this model we tested our hypothesis that the rough morphotype of *M*. *abscessus* will cause a greater host inflammatory response than the smooth morphotype. Preliminary results of this study have been previously published in the form of an abstract[22].

## Materials/Methods

### Animals

Wild-type mice on the C57BL/6 background were originally obtained from Jackson laboratories (Bar Harbor, ME). CF mice (S489X *Cftr* mutation with human CFTR gut correction via the fatty acid binding protein promoter, on the C57BL/6 genetic background) were originally obtained from Case Western Reserve University. Mice were ages 3–6 months, sex-matched and weighed 20–40g. Colonies were maintained under pathogen-free conditions within the Biological Resource Center at National Jewish Health. Experiments were conducted in a separate Animal Biohazard Facility within the Biological Resource Center.

### Ethics statement

This study protocol was approved by the **Institutional Animal Care and Use Committee of National Jewish Health** (permit number AS2791–06–14). All intratracheal inoculations were performed under anesthesia with 5% isofluorane, and euthanasia was performed with CO2 asphyxiation. All efforts were made to minimize suffering.

### Bacterial growth

American Type Culture Collection (ATCC, USA) fully sequenced strain 19977 of *M*. *abscessus* (subspecies *M*. *abscessus sensu stricto*, isogenic smooth and rough morphotypes from spontaneous conversion) was stored in 1 mL aliquots at-80C. Prior to infection one aliquot was thawed and then grown for 3 days in 25 mL of Middlebrook 7H9 broth (Difco, Becton, Dickinson and Company, Sparks, MD) supplemented with ADC (Becton, Dickinson and Company, Sparks, MD), glycerol and Tween-80 (Sigma-Aldrich, St. Louis, MO) at 37C with shaking at 200 rpm. On the day of infection the bacteria were pelleted by centrifugation at 3500 rpm for 10 minutes and washed twice in sterile phosphate-buffered saline (PBS). After the final wash the bacteria was suspended in 5 mL sterile PBS and further diluted to an optical density (OD) 650 of 1.2 for smooth and 0.9 for rough. Based on previously generated OD curves this delivered ~8 x 10^7^ colony forming units (CFU) of *M*. *abscessus* per mouse in 200 μL total inoculum fluid. CFU delivered were confirmed by plating serial dilutions of the bacteria on Middlebrook 7H10 agar (Difco, Becton, Dickinson and Company, Sparks, MD) supplemented with OADC (Becton, Dickinson and Company, Sparks, MD) and glycerol.

### Thrombin and fibrinogen solutions

Sterile thrombin (GenTrac Inc, Middleton, WI) was diluted to 1000U/mL with sterile PBS and stored in 500 μL aliquots at-20C. Immediately prior to inoculation, aliquots were thawed in a water bath at 37C, then diluted 5:1 vol/vol with sterile PBS at 37C and desired quantity of *M*. *abscessus* was added. Thrombin solution was stored on ice until time of inoculation. A saturated fibrinogen (MP Biomedicals LLC, Solon, OH) solution was made in sterile PBS at 37C and then diluted 2:1 vol/vol with sterile PBS at 37C before desired quantity of *M*. *abscessus* was added. The fibrinogen solution was kept at room temperature and used within 60 minutes of preparation. Sterile thrombin and fibrinogen solutions were used for the control groups.

### Infection process

**Fig 1:**
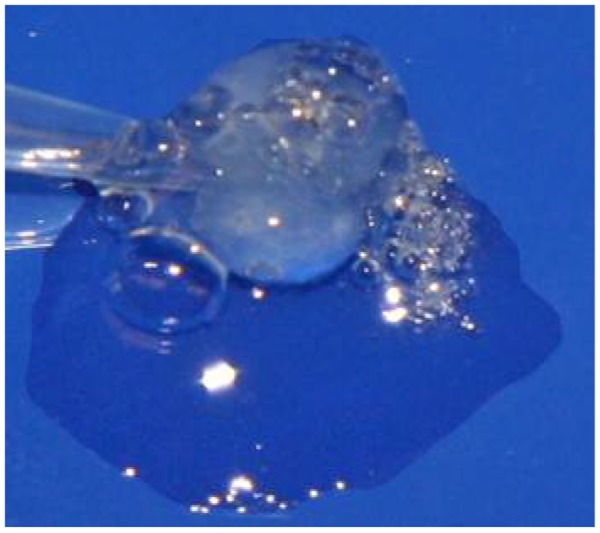
Fibrin plug model. Thrombin and fibrinogen solutions, combined here on the bench top, form gelatinous fibrin plugs that retain the bacteria in the distal airways.

Mice were briefly anesthetized with 5% isofluorane (Isothesia, Butler Animal Health Supply, Dublin, OH) and placed on a tilting rodent work table (Hallowell, Pittsfield, MA). The vocal cords were visualized with a rodent laryngoscope (Welch Allyn, Skaneateles Falls, NY) modified with a 7X surgical loupe attached for magnification. Under direct visualization a gavage needle with a 30 degree bend was inserted into the distal trachea and 50 μL thrombin and 50 μL fibrinogen were sequentially instilled, followed by a 150 μL air bolus to clear the needle and trap the bacteria in the distal airways in gelatinous plugs (Fig. 1). The mice were allowed to briefly recover to the point of regaining spontaneous movement then were reanesthetized and the inoculation process was repeated once. This resulted in ~8 x 10^7^ CFU of *M*. *abscessus* in a total of 200 μL of fluid delivered to the airway of each mouse.

The smooth and rough morphotype infections were performed separately, and did not overlap with each other. Two to three replicate experiments were performed for each morphotype. During each experiment 4–5 mice of each mouse type were infected for each planned sacrifice time point (total of 59 WT mice and 41 CF mice were inoculated with *M*. *abscessus*). Two replicate experiments with inoculation of sterile thrombin and fibrinogen were performed with 3–5 of each mouse type per sacrifice time point (total of 16 WT mice and 6 CF mice were inoculated with sterile thrombin and fibrinogen).

### Data collection

Mice weight trends were monitored. At days 3 and 14 days post-infection mice were euthanized via CO2 asphyxiation and direct cardiac puncture. Bronchoalveolar lavage fluid (BALF) was obtained with 1000 μL of sterile PBS plus protease inhibitor (Complete EDTA-free, Roche diagnostics, Mannheim, Germany) instilled into the lungs through a gavage needle inserted into the distal trachea, then removed with a syringe. A second lavage was performed with 500 μL of sterile PBS plus protease inhibitor in the same fashion. The lungs and spleen were then removed and placed in separate homogenization tubes with 700 μL sterile PBS plus protease inhibitor. BALF was plated on Middlebrook 7H10 agar (Difco, Becton, Dickinson and Company, Sparks, MD) supplemented with OADC (Becton, Dickinson and Company, Sparks, MD) and glycerol. Remaining BALF was centrifuged. The supernatant was stored in aliquots at-80C for future cytokine and chemokine analysis. The cell pellet was resuspended in 1 mL of sterile PBS, followed by analysis for leukocyte count with a Beckman-Coulter counter (Beckman-Coulter Inc, Miami, FL), then cytospun onto glass slides. The slides were stained (Hema 3 stain set, Fischer Scientific Company LLC, Kalamazoo, MI) and then analyzed with microscopy for leukocyte differential in a blinded fashion. Lung and spleen tissues were homogenized with autoclaved stainless steel beads in a Bullet Blender tissue homogenizer (Next Advance, Inc, Averill Park, NY), then serially diluted with sterile PBS and plated on Middlebrook 7H10 agar. Plates were incubated at 37C and CFU were counted at 5 days. Morphotype determination was made at 7 days.

### ELISA

ELISA was performed on BALF supernatant for KC, TNF-α, and IL-1β based on manufacturer’s instructions (ELISA Tech, Aurora, CO). Lower limit of detection for the ELISA kits was 0.01 ng/mL.

### Histology

Mice were inoculated with *M*. *abscessus* as described above. After euthanization, the lungs were inflated to 20 cm water with 4% paraformaldehyde for 15 minutes (Acros Organics, New Jersey, USA). The heart-lung block was then removed and submerged in 4% paraformaldehyde for 48 hours of fixation. The right inferior lobe was then removed, sequentially dehydrated and embedded in paraffin for sectioning, slide fixation and staining with H/E. Histology was blindly reviewed.

### Statistical analysis

To investigate the pattern of weight change over time, weight change from baseline was modeled as a function of mouse type (WT vs. CF) and infection type (rough vs. smooth) separately for mice sacrificed at day 3 and for mice sacrificed at day 14. Using a linear mixed model with a random intercept to allow for different initial weight changes and robust variance estimation, day from baseline was modeled as a categorical variable for mice sacrificed at day 3 and as a continuous variable for mice sacrificed at day 14. Additionally, in the model for mice sacrificed at day 14, a quadratic term of days and a random slope for days from baseline was included to allow for variation in the pattern of average weight change. There were 3 (CF) mice that were sacrificed at day 3 which were outliers and were excluded from the model. Weight data was analyzed with SAS software, version 9.3 (SAS Institute).

Unpaired t tests were used to compare the means of groups for the CFU and BALF data. Values for single comparisons were considered statistically significant at p<0.05. For multiple comparisons, a Bonferonni-adjusted *α* value (0.05/number of comparisons) was used. All mice that were sacrificed on their intended day were included in the analyses for the CFU and BALF data. Seven mice died at the time of inoculation due to airway obstruction from the fibrin plug. These mice were excluded from the analyses, as was the one mouse that died 4 days post-inoculation. Data are reported as mean +/- standard error of the mean (SEM). Data analyses and figures were generated with GraphPad Prism Software (version 6.02, San Diego, CA).

## Results

Intratracheal inoculation of mice with either smooth or rough morphotype of *M*. *abscessus* suspended in a fibrin plug consistently established pulmonary infection in both WT and CF mice. Of the 100 mice inoculated with M. abscessus, colony growth of *M*. *abscessus* from plating of lung homogenates occurred in all but one mouse (99%).Mortality rate was 2.6% (2/75) for WT mice and 12.8% (6/47) for CF mice, with nearly all mortality occurring at the time of inoculation from apparent airway obstruction by fibrin plugs.

**Fig 2:**
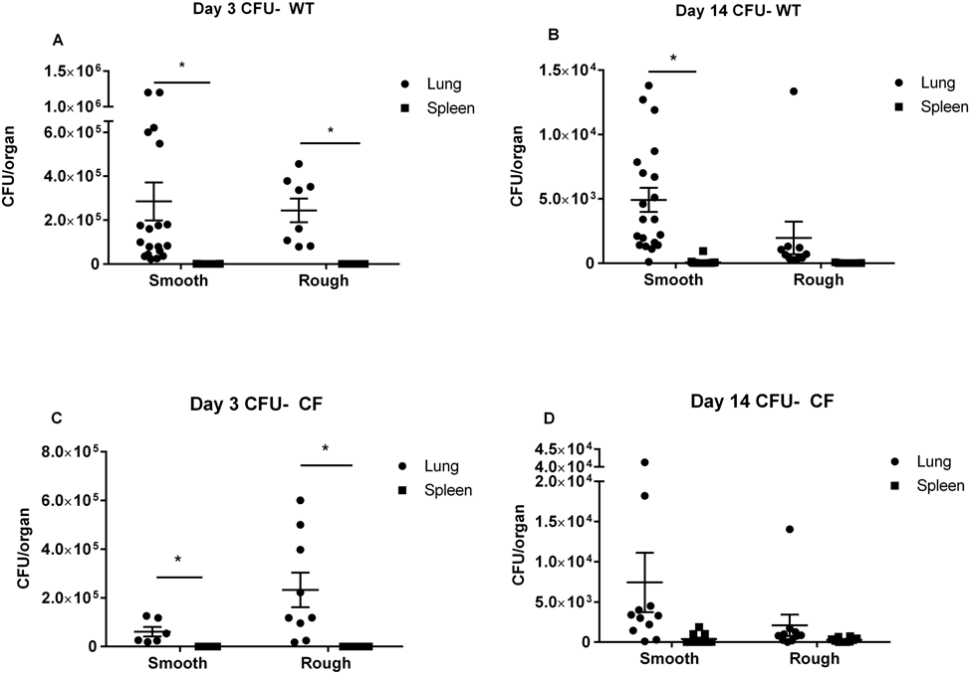
Pulmonary and splenic CFU. (**A**, **B**, **C** and **D**) Intratracheal inoculation with *M*. *abscessus* (smooth and rough morphotypes) suspended in fibrin plugs results in predominance of pulmonary infection with minimal systemic infection. (N = 9–20 mice in each group at 3 and 14 day time points). * p< 0.05. Data displayed as mean ± SEM of group, pooled from 2–3 replicate experiments for each morphotype.

The smooth and rough morphotypes created similar patterns of infection to each other, and pulmonary infection predominated over systemic infection (Fig. 2). In WT mice, significantly more *M*. *abscessus* CFU were recovered from the lungs than from the spleen with both the smooth (p = 0.0021) and rough (p = 0.0005) morphotypes at 3 days post-inoculation (Fig. 2). At 14 days post-inoculation in WT mice, significantly more *M*. *abscessus* CFU were recovered from the lungs than from the spleen with the smooth (p = <0.0001) morphotype. While infection with the rough morphotype did not meet statistical significance in WT mice at 14 days post-inoculation (p = 0.1652), pulmonary CFU were > 2 orders of magnitude greater than splenic CFU. In CF mice, significantly more *M*. *abscessus* CFU were recovered from the lungs than from the spleen with both the smooth (p = 0.0116) and rough (p = 0.005) morphotypes at 3 days post-inoculation. While infections with either the smooth (p = 0.0707) or the rough (p = 0.1916) morphotype did not meet statistical significance in CF mice at 14 days post-inoculation, pulmonary CFU were > 1 order of magnitude greater than splenic CFU for both morphotypes (Fig. 2).

**Fig 3:**
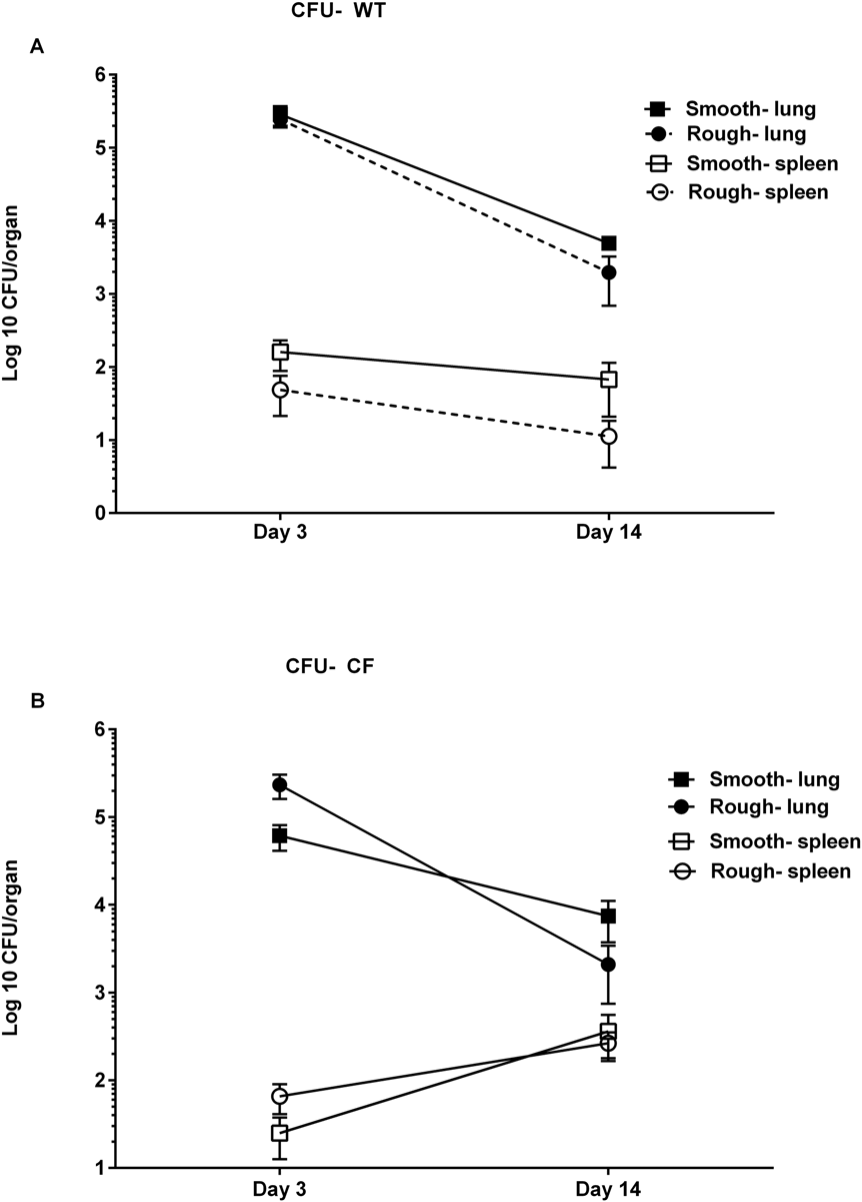
Changes in pulmonary and splenic CFU over time. (**A** and **B**) Pulmonary infection wanes over time with both smooth and rough morphotypes and in both WT and CF mice. Data displayed as mean ± SEM of group, pooled from 2–3 replicate experiments for each morphotype.

Pulmonary infection persisted over 14 days but the burden of infection waned over time. Numbers of pulmonary CFU trended downwards over time for both mouse types and infection types (Fig. 3). For both infection types splenic CFU trended downwards over time in WT mice but trended upwards over time in CF mice. Intratracheal inoculation with sterile fibrin plugs resulted in virtually no colony growth from either lungs or spleen at 3 or 14 days post-inoculation. Surprisingly, inspection of colonies on solid media from lung and spleen homogenates and BALF demonstrated that in 12/57 (21%) of the mice infected with the smooth morphotype, some colony conversion to rough morphotype occurred. Morphotype conversion occurred in both WT and CF mice and at both the 3 day and 14 day time-points. No smooth colonies were recovered from mice infected with the rough morphotype.

**Fig 4:**
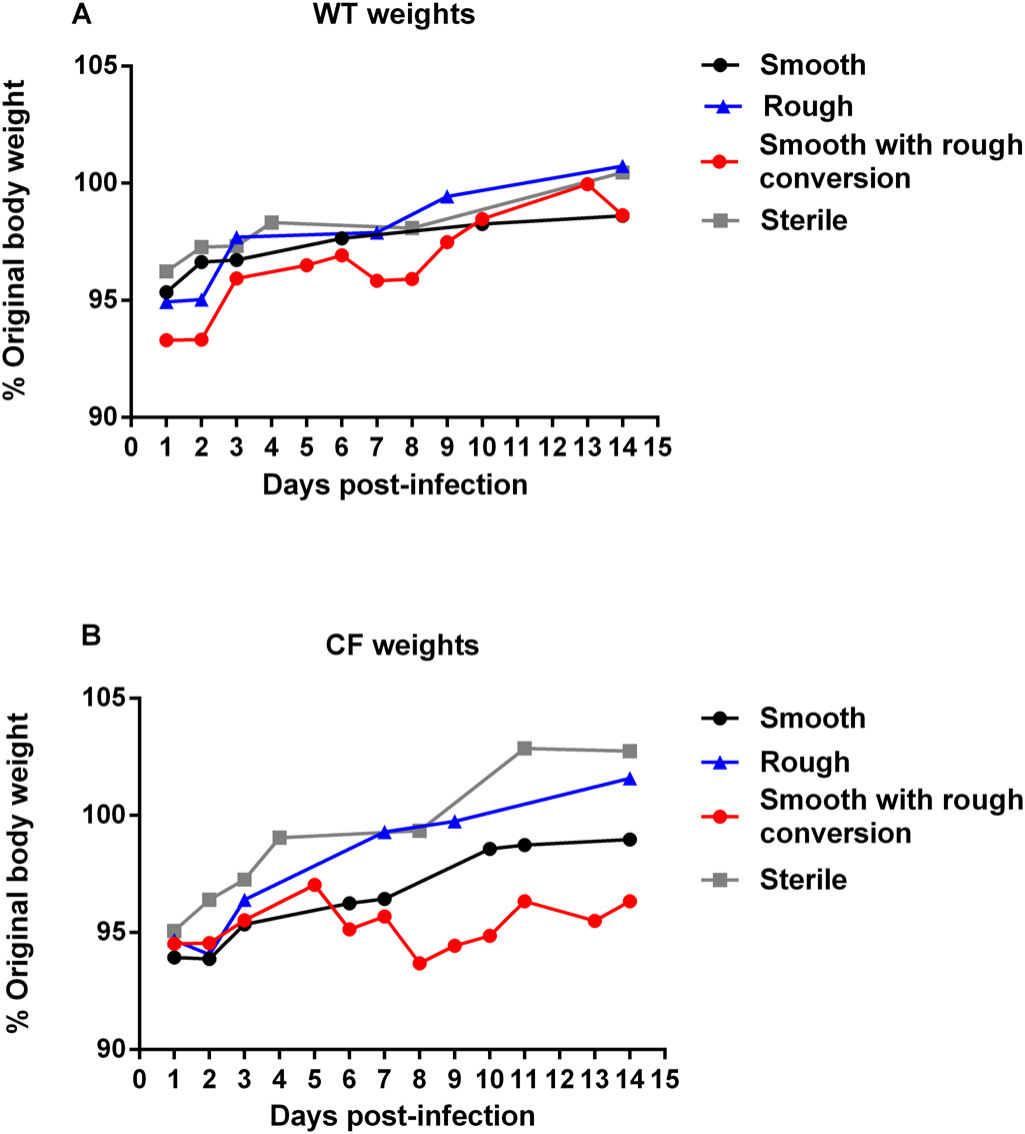
Weight trends. (**A** and **B**) Mice inoculated with *M*. *abscessus* trended towards greater weight loss than mice inoculated with sterile thrombin/ fibrinogen. (N = 19–40 mice in each *M*. *abscessus* group, N = 6–16 mice in each sterile inoculum group). Mice receiving smooth morphotype inoculation that had some colony conversion to rough morphotype on either BALF, lung, or spleen cultures had trend towards greater weight loss and slower weight gain. (N = 8–10 for WT mice, 4–6 for CF mice). Data displayed as mean of group, pooled from 2–3 replicate experiments for each morphotype.

Weight loss post-inoculation, overall, was minimal (Fig. 4). Weight change from baseline as a function of mouse type (WT vs. CF) was not statistically significant for mice sacrificed at day 3 (p = 0.073) or day 14 (p = 0.64). Weight change from baseline as a function of infection type (rough vs. smooth) was not statistically significant for mice sacrificed at day 3 (p = 0.13) or day 14 (p = 0.52). We did not find any significant interactions between days post-infection, mouse type or infection type. Both WT and CF mice trended towards greater weight loss with either morphotype of *M*. *abscessus* compared to inoculation with sterile thrombin and fibrinogen. Mice with some colony conversion from smooth to rough morphotype on culture experienced a trend towards greater weight loss and slower return to original body weight compared to the mice that maintained all smooth colonies. This trend was more pronounced in the CF mice compared to WT mice (Fig. 4).

**Fig 5:**
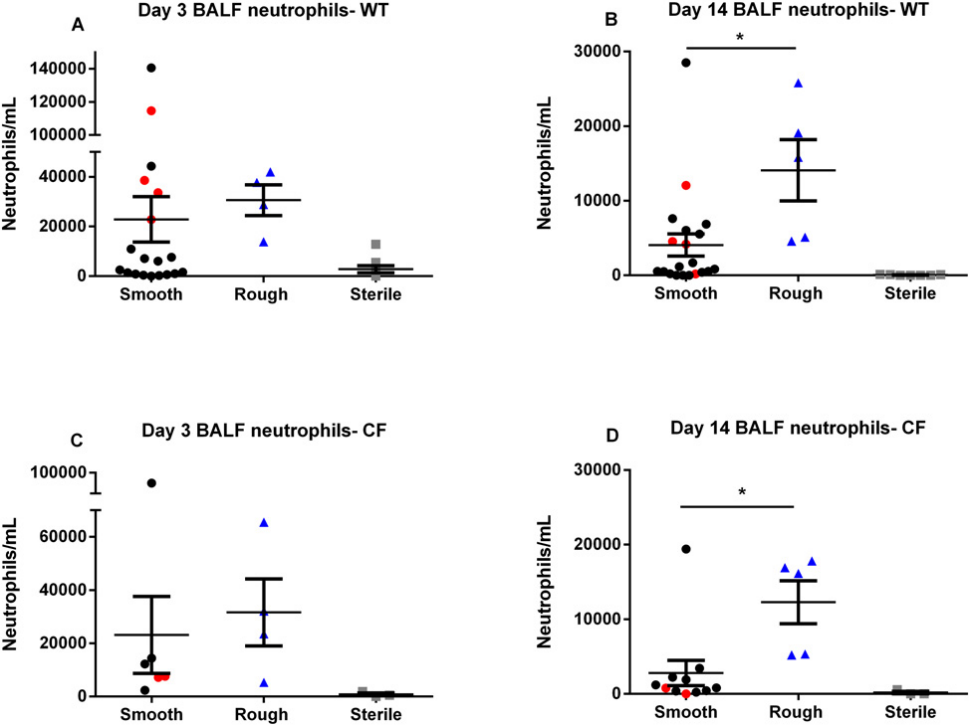
BALF neutrophils. (**A**, **B**, **C** and **D**) Inoculation with rough morphotype causes greater BALF neutrophilia than smooth morphotype at 14 days post-inoculation in both WT (**B**) and CF (**D**) mice. Red circles indicate smooth morphotype inoculation with conversion to rough morphotype. Minimal BALF neutrophilia was seen after sterile thrombin/fibrinogen inoculation. (N = 4–20 mice in each *M*. *abscessus* group, N = 3–8 mice in each sterile group). * p< 0.05. Data displayed as mean ± SEM of group, pooled from 2–3 replicate experiments for each morphotype.

**Fig 6:**
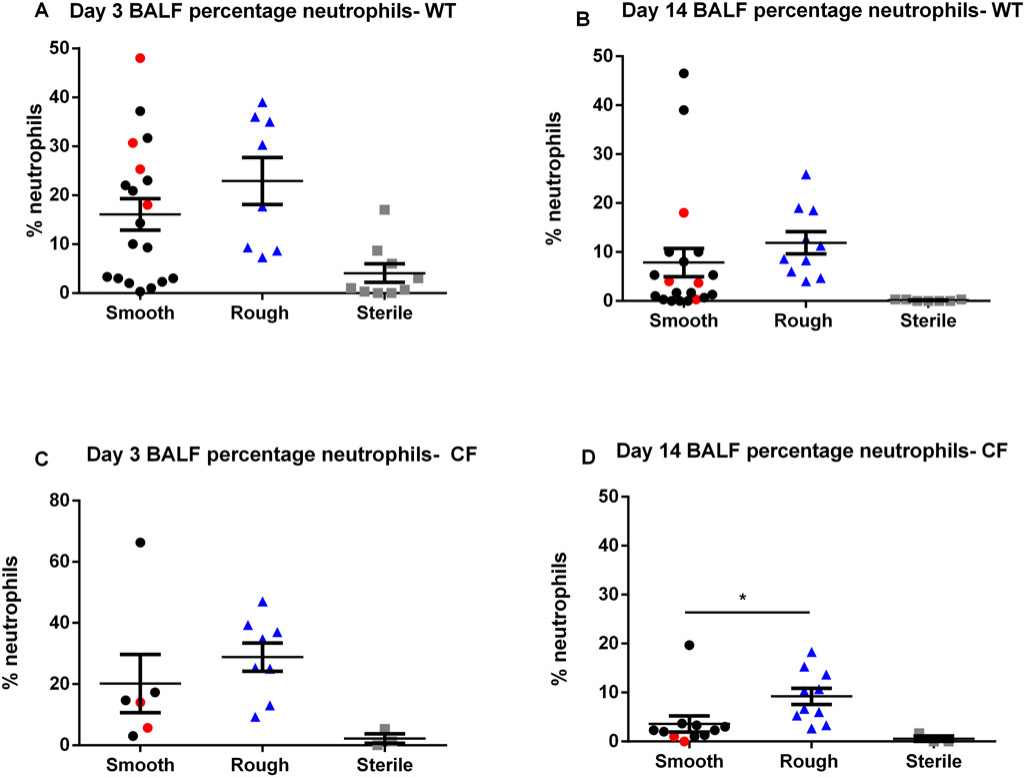
BALF neutrophil percentage. (**A**, **B**, **C** and **D**) Inoculation with rough morphotype causes greater BALF neutrophil percentage than smooth morphotype at 14 days post-inoculation in CF mice (**D**). Red circles indicate smooth morphotype inoculation with conversion to rough morphotype. Minimal BALF neutrophilia was seen after sterile thrombin/fibrinogen inoculation. (N = 9–20 mice in each *M*. *abscessus* group, N = 3–8 mice in each sterile group). * p< 0.05. Data displayed as mean ± SEM of group, pooled from 2–3 replicate experiments for each morphotype.

**Fig 7:**
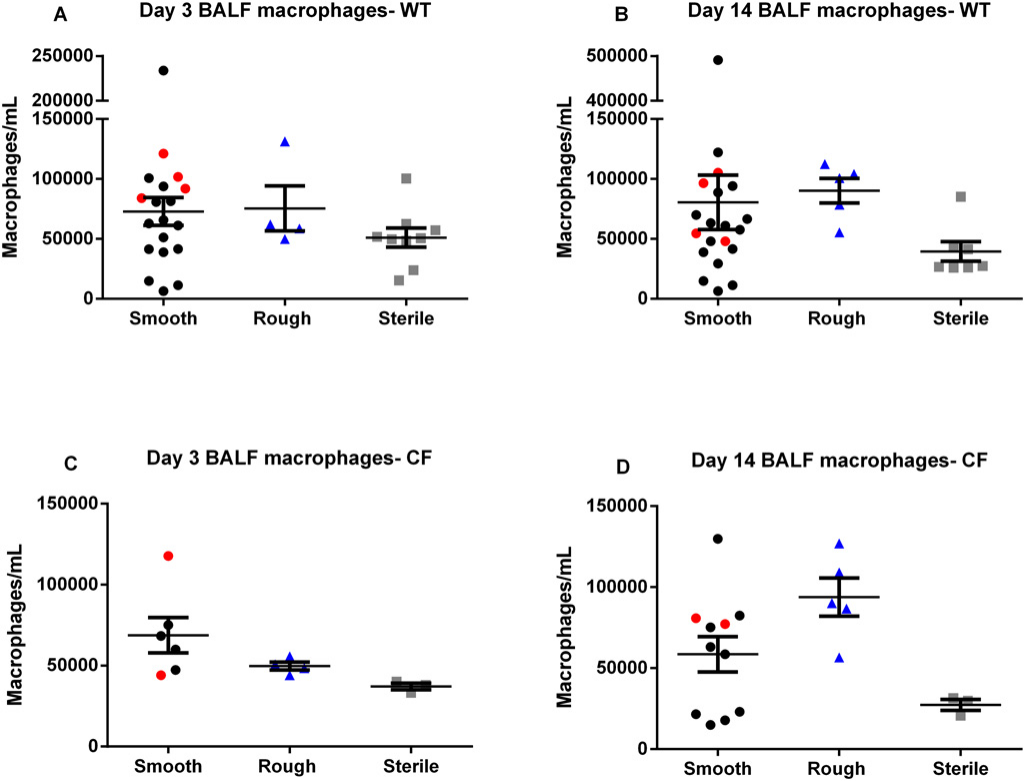
BALF macrophages. (**A**, **B**, **C** and **D**) Levels of BALF macrophages did not differ between groups. (N = 9–20 mice in each *M*. *abscessus* group, N = 3–8 mice in each sterile group). Data displayed as mean ± SEM of group, pooled from 2–3 replicate experiments for each morphotype.

Inoculation with the rough morphotype resulted in a greater pulmonary inflammatory response than the smooth, with significantly greater numbers of BALF neutrophils at 14 days post-inoculation in both WT (p = 0.0103) and CF mice (p = 0.0095) (Fig. 5). Inoculation with the rough morphotype resulted in a significantly greater percentage of neutrophils in the BALF cell count in CF mice at 14 days post-inoculation (p = 0.0271), and a consistent trend towards a greater percentage of neutrophils in the BALF cell count in CF mice at 3 days post-inoculation, and in WT mice at both time points (Fig. 6). Inoculation with the rough morphotype resulted in a trend towards greater numbers of BALF macrophages compared to infection with the smooth morphotype at 14 days post-inoculation in both WT and CF mice (Fig. 7). Numbers of BALF neutrophils and macrophages did not differ between the mice that were inoculated with the smooth morphotype but had some colony conversion to rough morphotype (red circles) and those that did not (black circles) (Figs. 5, 6 and 7).

**Fig 8:**
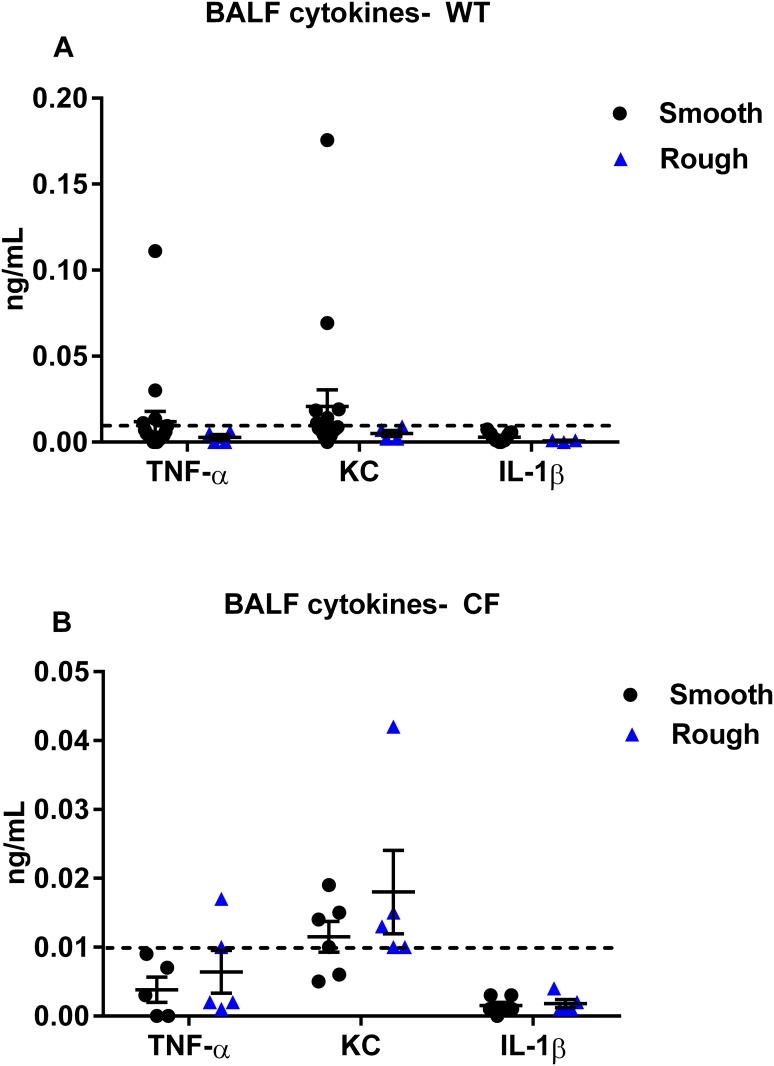
BALF cytokines. (**A** and **B**) BALF cytokines 3 days post-inoculation did not differ between groups. (N = 3–18 in each WT group, N = 5–6 in each CF group). Data displayed as mean ± SEM of group, pooled from 2–3 replicate experiments for each morphotype. Dashed line indicates lower limit of detection of kit.

BALF levels of TNF-α, KC and IL-1β were not significantly different at day 3 post-inoculation between the *M*. *abscessus* smooth and rough morphotypes in either WT or CF mice, and in the majority of cases were below the lower limit of detection of the ELISA kit (Fig. 8). In the WT mice there was a trend towards greater BALF levels of TNF-α, KC and IL-1β with the smooth compared to the rough infection, whereas in the CF mice this trend was reversed (Fig. 8). BALF levels of TNF-α, KC and IL-1β were not significantly different between the mice that were inoculated with the smooth morphotype but had some colony conversion to rough morphotype and those that did not (**data not shown**). Pulmonary histologic changes, including bronchiectasis, granulomas, or inflammation, were not found in any of the groups (data not shown).

## Discussion

We describe a murine model of pulmonary *M*. *abscessus* infection in which intratracheal inoculation of *M*. *abscessus*, suspended in sequential thrombin and fibrinogen solutions, traps the bacteria in fibrin plugs within the distal airways. Pulmonary infection with both smooth and rough morphotypes of *M*. *abscessus* was consistent across relatively large numbers of animals, including CF mice. While we achieved our goal of establishing a pulmonary infection with little systemic spread in immune competent animals, the burden of pulmonary infection had significantly decreased by 14 days post-inoculation. In our model, infection with the rough morphotype resulted in a great number of BALF neutrophils compared to the smooth morphotype at 14 days post-inoculation in both WT and CF mice.

Lack of a representative animal model has been cited as a major impediment to research on *M*. *abscessus* (and other NTM) pulmonary infections in diseases such as CF[21]. Previously, immune competent mice given an intratracheal inoculation of *M*. *abscessus* showed rapid clearance of the smooth morphotype from the lungs, and decreasing levels of the rough morphotype in the lungs within 14 days[10]. Persistent *M*. *abscessus* infection in mice can be achieved through the use of immune compromised mice and/or intravenous (IV) administration of the organism via tail vein injection, but the IV route of infection results in a systemic burden of infection greater than that seen in the lung[11,17–19], and elicits different host immune responses than inhalational infections[17,18,20,21].

Continued improvements to animal models of *M*. *abscessus* pulmonary infection are needed to help guide clinical research on the many questions surrounding this difficult infection in CF patients. Given the wide clinical spectrum of disease associated with *M*. *abscessus* infection in CF, along with the multiple comorbidities and multiple co-existing organisms in the their airways, it is often difficult to determine the contribution of *M*. *abscessus* to a patient’s clinical status[23]. Deciding if and when to initiate treatment for *M*. *abscessus* is further complicated by the treatment regimens, which consist of months of multiple antibiotics associated with significant costs and drug toxicities[24]. Currently, knowledge of *M*. *abscessus* virulence factors that may guide these difficult treatment decisions is lacking. Additionally, the increasing concern for patient-to-patient transmission of *M*. *abscessus* in CF further underscores the need to better define *M*. *abscessus* virulence factors, such as morphotype, to help inform prognosis and proper infection control measures[25,26].

We chose a fibrin plug as our vehicle for bacterial retention in the airways to improve the biological relevance of our pulmonary infection model. CF mice, including the S489X mutation mice used here, lack spontaneous development of pulmonary infection, airway mucus obstruction, or bronchiectasis[27,28]. Thus CF mouse models of chronic infection have relied on artificial means of retaining bacteria in the airways, such as agar beads, to create prolonged pulmonary infection[29–31]. Fibrin is found in airways inflammation[32], and is a component of obstructive airway mucus plugs and casts in several pulmonary diseases known to be susceptible to NTM infections, including cystic fibrosis[33]^,^[34,35]. Intratracheal inoculation of bacteria suspended in a fibrin plug has been successfully used to create a prolonged *Pseudomonas aeruginosa* pulmonary infection in mice [36]. Despite the inherent differences between human and mouse CF pulmonary manifestation, CF mouse models of *M*. *abscessus* and other infections have proven useful for evaluation of host immune response to infections and in testing drugs and novel therapeutics[11,17,18,21,28,36].

While the clinical relevance of *M*. *abscessus* morphotype distinction remains unclear, our findings support a growing body of evidence from cell culture systems and animal models of *M*. *abscessus* infection that the rough morphotype causes a greater host inflammatory response than the smooth[10,11,19,37,38]. In our model, infection with the rough morphotype of *M*. *abscessus* resulted in a greater neutrophil-rich inflammation compared to the smooth morphotype 14 days post-inoculation in both WT and CF mice. Neutrophilic airway inflammation is an important pathogenic mechanism in CF lung disease, and for instance has been associated with reduction in lung function in infants with CF[39].

Though greater neutrophilic airway inflammation resulted from inoculation with the rough compared to the smooth morphotype at 14 days post-inoculation, the overall degree of pulmonary inflammation found with either *M*. *abscessus* morphotype infection was modest, and decreased over time. The relatively low level of pulmonary inflammation in the setting of *M*. *abscessus* infection is consistent with our prior report, in which human neutrophils released lesser quantities of proinflammatory cytokines when stimulated by *M*. *abscessus* compared to *Staphylococcus aureus*[40], and is consistent with our knowledge of the often indolent course of *M*. *abscessus* infection in CF patients. The overall modest inflammatory response to *M*. *abscessus* infection with either morphotype is reflected in the low levels of BALF cytokines 3 days post-inoculation. Prior models have demonstrated BAL cytokines peaking at 3 days post- *M*. *abscessus* infection, then decreasing by day 7[18]. We thus did not test BALF cytokines at the 14 day post-inoculation time point given that the majority of the 3 day results were below the lower limit of detection of the ELISA kits, as we expected cytokine levels to be undetectable at the 14 day time point.

In the described model the burden of pulmonary infection based on CFU from lung homogenates decreased between the 3 and 14 day time points. This model characteristic likely represents a limitation of the model. It is worth noting, however, that *M*. *abscessus* infections often exist at a lower burden of infection in the CF lung than other typical CF pathogens. In Olivier’s multicenter prevalence study of NTM in CF, only 26% of patients who were culture positive for NTM were also smear positive[4]. A positive NTM smear requires a minimum bacterial load of 10^3^ organisms/mL[41]. Thus the majority of CF patients with NTM likely have an NTM bacterial load less than 10^3^ cfu/mL, which is in contrast to a recent report in which the mean density of *Pseudomonas aeruginosa* in CF sputum samples was >10^7^/mL[42]. Though the waning pulmonary infection over time will likely limit the utility of this model in applications that require prolonged infection, such as drug efficacy studies, the ability of the described model to consistently create a low burden of pulmonary infections at 14 days post-inoculation may be useful for testing differences in bacterial virulence and host response. For instance, it was with this reduced (compared to 3 days post-inoculation) burden of pulmonary infection at the 14 day time point that we observed the significant increase in BALF neutrophil levels related to the rough compared to the smooth morphotype infection.

We also observed, to our knowledge, the most consistent *in vivo* conversion of *M*. *abscessus* from smooth to rough morphotype, with morphotype conversion occurring in 21% of mice inoculated with the smooth morphotype. While significant differences were not seen between the mice that were infected with the smooth morphotype and had some *in vivo* conversion to the rough morphotype compared to those mice that maintained all smooth colonies on culture of lung, spleen, and BALF, this analysis was limited by group size demonstrating *in vivo* conversion. We acknowledge the possibility that the initial *M*. *abscessus* inoculum contained both smooth and rough morphotypes in the mice that appeared to have *in vivo* colony conversion. However, serial dilutions of each smooth morphotype inoculum were plated on Middlebrook 7H10 agar on the day of inoculation, and only smooth colony growth was observed on these plates, providing supporting evidence for the morphotype conversion having occurred *in vivo*. While recent work has identified genetic changes responsible for the smooth to rough conversion, the factors that influence morphotype conversion remain unclear[43].

Finally, minimal histological changes were seen in the mouse lungs after infection with either morphotype. Lack of pulmonary histological changes in the setting of infection with the smooth morphotype is consistent with Byrd’s finding in the original mouse model comparison of *M*. *abscessus* morphotypes[10]. Similarly, in a more recent aerosol infection model of *M*. *abscessus* (morphotype not described) in C57BL/6 mice, pulmonary infection was achieved but minimal pulmonary histologic changes were seen [18]. However, the lack of pulmonary histological changes that we observed in the setting of rough morphotype infection differs from prior reports[10]. In Ordway’s aerosol model of infection with *M*. *abscessus* rough morphotype, both WT and immune compromised mice had peribronchiolar inflammatory infiltrates on lung histology 15 days after infection that were worst in the lower lobes[17]. We thus performed our analysis at 14 days post-inoculation on the right lower lobes of the mice. While we expect to have seen histological changes at this time point and location if they were to occur, we do acknowledge the potential for histological changes to have occurred in different locations and/or at different time points. Additionally, our use of a fibrin plug as a mechanical means of retaining the infection in the airways likely alters the bacteria’s ability to form biofilms and invade host tissue, which may account for the lack of histologic changes seen in our mice with rough morphotype infection.

While the lack in any of these models of the caseous necrosis and granulomas that are considered to be characteristic of human NTM pulmonary disease[44] may reflect remaining inadequacies in current mouse models, including ours, there is evidence that *M*. *abscessus* pulmonary infections inconsistently cause histologic changes in CF patients as well. In an autopsy study of CF patients with positive NTM culture, pulmonary granulomas were present in only 2 out of 6 CF patients with multiple positive NTM cultures, and no histologic changes characteristic of NTM infection were present in 12 patients who had a history of a single positive NTM culture[45].

In addition to the lack of pulmonary histological changes, we recognize that this model has other significant limitations in its applicability to human disease. It remains an artificial mode of *M*. *abscessus* infection with a single intratracheal inoculum that differs from the repeated small inhalational exposures that people likely encounter in the environment. Similarly, infection with *M*. *abscessus* in isolation differs from the polymicrobial infections traditionally found in CF. The small number of surviving mice, particularly of CF mice, at certain time points limits the power of our analyses and likely contributes to the lack of statistical significance in many of the outcome measures. However, despite our small numbers we did find statistical significance in several important outcome measures demonstrating both the success of the model in establishing a predominance of pulmonary over systemic infection with both morphotypes and in increased pulmonary neutrophilia from the rough compared to the smooth morphotype.

The described model both highlights the continued difficulties of establishing a representative animal model of pulmonary *M*. *abscessus* infection, as well as offers an alternate model with some characteristics that represent improvements (albeit small) on existing models. Despite recognized limitations, the ability of the animal model described to achieve consistent pulmonary infection in immune competent mice with minimal systemic infection may improve the ability to research *M*. *abscessus* virulence factors and host-pathogen interactions. Based on evidence from prior animal and cell culture models of increased virulence from infection with the rough morphotype, and supported by our findings of an increased neutrophil response with rough compared to smooth morphotype infection, morphotype identification offers a potential marker of *M*. *abscessus* pathogenicity. While our results to date clearly do not answer the question of the role of morphotype in *M*. *abscessus* disease course, they do reinforce the need for clinical studies to test the potential role morphotype differences play in *M*. *abscessus* pulmonary disease. Though identification of *M*. *abscessus* morphotype is uncomplicated and could be easily done in clinical laboratories, more information on the clinical relevance of morphotype differences is needed prior to implementing widespread changes in laboratory reporting practices. We hope that this model will be useful for further investigations of *M*. *abscessus* virulence factors and host-pathogen interactions, and that continued improvements to this and other animal models will be realized to more closely represent what is observed in human diseases such as CF.
